# Academy of Stomatology

**Published:** 1895-03

**Authors:** George D. B. Darby

**Affiliations:** Secretary


### ACADEMY OF STOMATOLOGY.

A regular monthly meeting of the Academy of Stomatology was held at the rooms of the Academy, 1731 Chestnut Street, Philadelphia, January 8, 1895, the President, Dr. Jack, in the chair.

After the transaction of routine business, Dr. S. H. Guilford presented to the library of the Academy certain publications donated by Mr. Charles F. Wells, son of the late Dr. Horace Wells, of Hartford, Conn., and said, "At the meeting recently held in Philadelphia to celebrate the Fiftieth Anniversary of the Discovery of Anæsthesia, Mr. Wells stated that he had in his possession certain documents of his father's that were out of print, and very kindly offered to send me copies of such as he had. I have received and desire to present the following publications to the library in the name of Mr. Charles F. Wells: 'Dr. Wells's Discovery of Anæsthesia,' published in 1870; 'The Discovery of Horace Wells,' 1850; and an 'Essay on the Teeth,' by Horace Wells, 1838."

The secretary presented a specimen consisting of an unusually large deposit of tartar attached to a dental plate, donated to the museum by Dr. Ford, of Toulouse, France. By vote of the Academy the secretary was requested to convey the thanks of the Academy to the donors.

The paper of the evening was then read by Dr. E. W. Stevens, of Philadelphia, on the subject "Cancer of the Tongue."

(For Dr. Stevens's paper, see page 146.)

### DISCUSSION.

*Dr. H. H. Burchard.*—Dr. Stevens states in his essay that heredity appears to play a small part in the origin of carcinoma. I remember Professor S. W. Gross placed the percentage as a low one; but there does appear to be a special diathesis connected with carcinoma, for all who are subjected to the same sources of local irritation are not victims, and in several individuals some local disease process may arise and show for a time the same conditions, but in one carcinoma results and in others does not. Carcinoma itself is a proliferation of epithelium in lymph-spaces and lymph-tracts; thus, the anatomical condition separating benign and malignant growths is that structure indefinitely described as basement membrane, a membrane composed of skin or gland, that being the barrier between the lowest layer of epithelium and lymph-spaces. It does not seem inclusive enough to ascribe, as Cohnheim does, the origin of leukoplasis to nests of embryonic cells, but undoubtedly this is a rational explanation in some cases. The present hypothesis of microbic origin is by no means conclusive.

In the invaginated epithelium of the embryonic jaw, which forms the enamel-organ, we see the sharp line of demarcation between epiblastic and mesoblastic tissue, and see how the line remains unbroken; but why epiblastic tissue should break from its restraint and proliferate in the spaces of mesoblastic tissue, is one of the most vaguely comprehended problems of pathology.

I have heard Dr. Stevens and other surgeons express themselves as sceptics in regard to the value of microscopic diagnosis of neoplasms, but I have the records of two cases to add as supplemental to his, which will serve to demonstrate that clinical diagnosis may be equally undeterminate; in fact, it scarcely needs argument to prove that it must be more so.

The first, a gentleman of about fifty years, was referred to me by the late Uriah Kirk. The patient had upon the left lateral aspect of the tongue, about midway, an ulcer, say one and a half inches long, one inch broad, elliptical; hard, raised edges, almost indurated, gray, pasty base, and giant granulation; shooting pains along the inferior maxillary nerve, but no lymphatic involvement.

He denied syphilis, and no history even pointing to that malady could be elicited. I extracted the stump of the wisdom-tooth, rounded and smoothed the lingual cusps of the second molar, gave him a salicylic acid mouth-wash, stopped his smoking, and in three weeks recovery followed.

I was almost certain that the disease was epithelioma; in fact, a surgeon had so diagnosed it. My treatment was designed as tentative, pending a consultation with Professor Garretson.

The second case, a gentleman for whom I was about to do dental work, spoke to me of an angry-looking ulcer on his tongue; this was about the size and had the appearance of the preceding one, except that it was more circumscribed, and the induration and giant granulations more pronounced. A surgeon had been making applications of nitrate of silver. There was no family history of carcinoma, but little induration, no lymphatic involvement and no pain. No history of syphilis. He desired that I treat him. The teeth were smoothed, tobacco and spirits prohibited, and potassium iodide, eight grains three times a day, prescribed. In ten days no change whatever, and I referred him to a surgeon. One-third of the tongue was amputated; sections demonstrated the disease to be epithelioma. The wound healed well, but in three months the disease recurred, this time appearing on the other side of the tongue, and in a few months death resulted.

In this case were none of the symptoms of carcinoma other than giant granulations, and yet it proved to be cancer; in the other were the symptoms and appearance of malignancy, and it proved to be benign.

*Dr. M. H. Cryer.*—I have very little to say on the matter of the paper, as I coincide almost entirely with the views expressed. I would like to ask Dr. Stevens what was the mouth-wash used in the case of which he spoke. He spoke of putting him under specific treatment, using a mouth-wash.

I infer that he makes no line of demarcation whatever between ulcer of the mouth and carcinoma; he also recommends surgical interference at a very early period. If there is anything in the germ theory, and we have an ulcer that forms a ground or nidus for germs to take root and produce a growth, if we should amputate, do we not make more ground or more of a sore in which the germs may proliferate, and would not death ensue much sooner?

In regard to the use of nitrate of silver, I have had a little experience in patients where it has been used, which have been sent to our hospital. By discontinuing the use of nitrate of silver, and putting the patient under other treatment, using a wash or the powder of subnitrate of bismuth, I have seen the cases much benefited.

*Dr. James Truman.*—This matter is of considerable importance to dentists, although I do not profess to know much about it, as very few of us have anything to do with cancer of the mouth, especially of the tongue. But, passing along through life, I necessarily have come across a number of such cases, and I have been impressed with one feature in relation to epithelial cancers,—viz., the tenacity with which many so afflicted hold on to life.

Ordinarily, in tumors of the mouth, I think it is very difficult to distinguish between those which are benign and those which are malignant. I had a patient that had a benign epithelial growth in the mouth for years. He wanted to know my opinion about a surgical operation, and I advised against it; and throughout his life it never gave him any further trouble.

Another case that came into my hands was decided epithelial cancer. As I would not think of treating such a case in the ordinary way, I sent him to a surgeon who operated for its removal. I saw the patient afterwards, and he was doing very well, but how long will that individual be likely to live? The growth will recur in ninety-nine cases out of a hundred.

Another case of epithelial cancer, not on the tongue, but on the lip: the man who had it outlived two of the surgeons that operated on it; both died; he was then in care of the third one. The entire anterior portion of the jaw was gone when I last saw him. He died, however, in the course of two or three months.

It seems to me that we, as dentists, are not careful enough in our treatment to remove all rough places from the mouth. I am one of those who believe in heredity. I believe that persons may have alteration of the tissue in such a way that it may easily become deranged by any irritation that may take place upon its surface. Of course I know that the general idea is that phthisis and all other diseases of that kind are not and need not be necessarily caused by irritants; that it is simply derangement of the tissue; the same thing occurs, I think, in cancerous affections.

*Dr. Stevens.*—The wash which I usually use in these cases, is a solution of ten grains of chromic acid to the ounce of water. It seems to remove pain and act beneficially. The treatment was employed, and the wash that was given, was simply a solution of borax and boracic acid.

In regard to the question raised by Dr. Cryer, as to amputating some of the tongue and furnishing a nidus for a new growth of carcinoma, it must be said that the etiology of cancer in the cases reported in the paper has remained down to the present day one of the most obscure problems of medicine. Lately some investigators thought they had discovered the cause in a parasite, but the idea was not realized and was vigorously opposed and in the end defeated. Up to the present time no specific microphyte, bacteria, or micrococcus has been cultivated from carcinomatous tumors. Neither have they been able to transfer the disease by bacteriological culture to any of the lower animals. It must be admitted, however, that many pathologists still hold the idea that cancer is of a parasitic nature, and there are strong clinical reasons for believing it to be an infective disease.

The literature on the subject is enormous, but the question has not been settled. I thought it would be better to leave that question out of the paper entirely. You will understand that a paper upon so large a question as this is simply touching upon the surface of a very large subject. The members of the dental profession are the ones who will have the best opportunity to observe these various conditions which I have endeavored to describe. There is a good deal to be learned about it, and it is only by observation that we can finally procure much information about it. The question of leucoma of the tongue is something not very well known to medicine. It is only of late years leucoma has been regarded as a predisposing cause of cancer. Many surgeons regard it as more significant of cancer than ulcer of the tongue. I have seen the condition several times myself; two cases referred to me by Dr. Jameson. One case a few weeks ago of syphilitic origin, undoubtedly; for the patient suffered with other symptoms of tertiary syphilis which quickly responded to proper treatment.

*Dr. Truman.*—Why do you use chromic acid instead of nitrate of silver?

*Dr. Stevens.*—Nitrate of silver seems to act as an irritant. All strong caustics should be avoided. It does very well in ulcers of young people, especially children; but experience has shown chromic acid to be more beneficial.

*Dr. Truman.*—The reason I ask is that, in my experience with chromic acid, it burns absolutely the albumen of the tissue; whereas nitrate of silver penetrates very deeply, coagulating very deeply, and I wondered whether that had anything to do with why chromic acid should be preferred to nitrate of silver. It seems to me chromic acid, because of its burning superficially and destroying the tissue, would be preferable to nitrate of silver.

*Dr. Stevens.*—Chromic acid acts as a superficial caustic, and those who are working in diseased conditions of the nose very well know there is a great deal of defective information regarding the use of chromic acid. It is a superficial caustic.

*Dr. Kirk.*—Will Dr. Stevens describe the characteristic features of the condition known as leucoma of the tongue somewhat more fully than he has in his paper?

*Dr. Stevens.*—I endeavored to have a couple of patients here to-night, but, as they were both specific cases, they did not care to be exhibited. Mr. Rau made photographs for me of these cases, but, as they do not show the condition, I thought best to leave them at home. Leucoma is really a superficial inflammation of the tongue,—superficial chronic glossitis. The tongue loses the natural papillæ, and becomes glassy and smooth. It is not a coating of the tongue or anything like a fur on the tongue, as we frequently see, but it is an affection of the superficial mucous membrane, and is soft. As I stated in the paper, it looks as if it had been brushed over with a layer of nitrate of silver.

*Dr. Kirk.*—Does it extend beyond the tongue, or encroach upon the rest of the mucous membrane?

*Dr. Stevens.*—It may extend from the tongue to the other mucous membrane, particularly that of the cheeks where the teeth come together, opposite the junction of the tongue and teeth, and may sometimes be observed on the gums also. It is very much like smoker's patch. In the mouths of those who smoke a pipe and never change its position in the mouth, letting the smoke come in contact with the same place, the tobacco smoke acts as an irritant, and produces local inflammation of the tongue, which is practically leucoma of the tongue; it is, however, of a darker color, and usually, in the cases I have seen, the coating peels off, leaving a raw surface exposed; but this condition known as smoker's patch is the same condition as that known as leucoma of the tongue.

*Dr. Truman.*—Does it not amount to ulceration underneath?

*Dr. Stevens.*—It predisposes the tongue to ulceration. In some places this leucoma peels off and leaves an open patch, which after a while becomes covered over again.

*Dr. Cryer.*—About eight years ago a patient came under my charge with what was then pronounced a carcinoma of the tongue, and it was the intention to have an operation performed. It was recognized by Professor Garretson as leucoma of the papillæ of the tongue and cheek. It commenced about the median line of the tongue, and extended over the surface of the left alveolo-lingual border into an open space where a second and third molar had been extracted on to the cheek, covering the whole region to the line of Well's duct, and it had, especially on the cheek, the glassy appearance Dr. Stevens described, and it was thought that at that point it might become malignant; for that reason the operation was suggested. Instead of the operation, the patient was placed upon a hygienic diet, and nothing was used on the parts except nitrate of bismuth painted over with a camel's-hair brush. The patient commenced to improve, and within six months had perfect comfort. He comes to the hospital every three months, and if there is a little patch having an angry look it is simply painted over with nitrate of bismuth. It is now, I believe, eight years that the patient has been thus carried along comfortably.

Another patient reported two years ago with very similar conditions; after treatment of about three months he left the hospital, and we have not heard from him since, and presume he is well; he was apparently quite well when he left.

*Dr. Jameson.*—During the last four years I have referred three patients, one to the late Dr. Agnew and two to Dr. Stevens. The first was fifty years of age, and, while doing dental work for him, I noticed he had an angry-looking sore on the median line of the lip. He was an inveterate smoker, but I cannot say this had anything to do with it.

I asked the patient how long he had been suffering with the sore, and he said several months. Being a friend, and being interested in him, I recommended him to go to one of our surgeons at once. He seemed to think it was of no importance, and went to Atlantic City, and in three or four months' time it gave him increased trouble. Dr. Reed and the late Dr. Agnew removed a portion of the lip. He came to have some dental work done some time after, and there was a small portion that had not healed; and owing to this I had considerable difficulty in doing the work. A second operation was afterwards performed, more of the lip being removed. He had three operations performed altogether, and in the course of two and a half years from the time I called his attention to the sore he died.

I think the function of the dentist is to be on the lookout for these conditions and recommend the cases at an early period to a specialist. In one patient, thirty-five years of age, I had noticed, instead of the glassy appearance Dr. Stevens describes, an appearance more like the cicatrix from a burn, although it did not have that color, but something more like lead color. There was also the same condition upon a line running antero-posteriorly to the lip where the cheek conforms to the teeth. I referred the case to Dr. Stevens, and he was put under treatment, and in a short time his tongue improved. Two of the most prominent surgeons of Philadelphia recommended that the tongue be removed. It was like a thunder-bolt out of a clear sky, and upset him very materially He put it off, and made such improvement under the treatment that it was not considered well to have the operation performed, and to-day he is better and more comfortable than for many years.

Four months ago I referred a gentleman from Chicago to Dr. Stevens, and upon seeing him a short time ago he reported that he had not been as well for a number of years. He had been treated in New York with nitrate of silver, and had gotten worse, but under the chromic acid and antisyphilitic treatment is now a comparatively well man. In both cases the men were very thankful that they had escaped the surgeon's knife.

On motion of Dr. Truman, a vote of thanks was tendered to the essayist, and the subject was passed.

CASUAL COMMUNICATIONS.

*Dr. Burchard.*—The revival of the interest in the uric-acid origin of phagedenic pericementitis, due to the essay of Professor Peirce, in which he demonstrated a relationship between the two conditions, has led most of us, no doubt, to a more careful search for arthritic history of patients who are the victims of pyorrhœa alveolaris.

In some fifty cases which I have seen, among my own patients and those of other dentists, there has been found an undeniable history of gout in more than half. In some were marked hereditary manifestations and acute outbreaks; in others, a rheumatic history. Some, in whom no evidence of what the text-books of medicine describe as even obscure gout, were unmistakably the subject of the American gout, lithæmia, the type of disorder graphically described by Da Costa in his "Medical Diagnosis."

It has been denied that the origin of the dental disease is ever found at any point other than the gingival margins. Professor Peirce's descriptions show the contrary; and, to add to his list, I have just seen a patient of Dr. Gilliams, who had the type of teeth which fall victims to this disease,—dense tissues, dental, alveolar, etc. Over a superior lateral and central incisor, near the apices of the respective roots were swellings, apparently abscesses; marked fluctuation at the summits of the swellings, and some turgescence of surroundings, but at the gingival margin the dental ligaments were undetached, and for half an inch above there was no evidence of inflammation. On incision through each swelling a free flow of blood followed, but apparently no pus; unfortunately, my microscope was not at hand, and this question remained undetermined, the blood flowing as freely as though a small vein had been incised. Passing a small scale through the openings made, there was found a loss of alveolar process and a marked roughness of the roots.

The patient had been seen some time before, and tartarlithine prescribed, and the swellings which were present disappeared.

The swellings reappeared exaggerated, coincident with an attack of acute muscular rheumatism. A rheumatic family history was obtained; a sister of the patient, a rheumatic, had lost several teeth through this process.

It seems that the results of the investigations as to this disorder must lead to renewed inquiry by the general pathologist; for this disease, associated as it is frequently and unquestionably with true gout, the arthritis of classical symptoms, the same dental manifestations are present in a series of disorders, most of which are described by Haig, in which an antigout treatment removes the general symptoms and ameliorates those relating to the teeth. The general manifestations are chronic gastric catarrh, hepatic disorders, forms of anæmia, renal diseases, and catarrhs in general; in all of which there is undoubtedly a close association with uric-acid retention.

I desire also to make a record of my experience in the use of salol.

Since the publication of Dr. Mascort's excellent paper in the *Dental Cosmos* on "Salol as a Root-Filling," I have been using that substance almost exclusively for this purpose, and, so far as some ten months of trial can prove, with uniform success. A solution of sodium peroxide is first used to saponify fatty material, dissolve and drive out the contents of the tubuli. This is neutralized by a weak solution of sulphuric acid followed by thorough drying with alcohol and hot blast; then with a pair of long-pointed dressing pliers a portion of crystals is taken up and held above a small flame until it becomes fluid. The closed points are then placed as high up the canal as possible, and slowly opened, the fluid runs up the dry canal. An iridium broach warmed is used to pump the salol to the apex, and in the still fluid material a point of metal or gutta-percha is thrust. This is used in the event of reopening of the tooth ever becoming advisable. By warming the point it may be withdrawn, and with it the melted salol, rendering access to the apex easy. Experimenting with teeth out of the mouth has demonstrated some difficulty in removal where a central mass of other material has not been used.

*Dr. Kirk.*—I would like to bear testimony to the value of salol as a root-filling material. Since the publication of Dr. Mascort's paper and for a month previous to that, because I had the paper in my possession fully a month before it was published, I have used nothing but salol for filling roots in my practice. I have, in some cases, used it in connection with a gutta-percha point, but generally without it. It crystallizes solidly in the canal. When it is melted and introduced into the warm, dry canal, it apparently flows into the uttermost extremities of it. It has a fluid character like melted paraffin or the paraffin oils, and in a few moments it crystallizes. I have yet to have the first case of apical pericementitis follow a case so treated, although I have been almost, you might say, careless in the use of it,—that is, I have used it in immediate treatment of cases and those of recent devitalization, and it has been uniformly satisfactory to me.

*Dr. Roberts.*—Salol melts at a very low temperature, so low that a mouthful of hot water will melt it if held there. Now, if it is melted by the use of hot drinks, would there be any absorption? Or is it dissolved in any way in water or moisture? And also, if it should melt, would there be any damage done in the canal?

*Dr. Kirk.*—It is quite impossible to melt salol in the root of the tooth with hot liquids taken as drink. It melts at 104° F., so that the danger suggested by Dr. Roberts would be eliminated, and it is only slightly soluble in water. A fluid at 107° F. would drop very quickly in temperature when taken into the mouth.

*Dr. Roberts.*—Slightly tepid water would melt salol, and some women drink tea very hot. You think a tooth would not carry the heat from a mouthful of hot water up so as to melt the salol?

*Dr. Kirk.*—"Slightly tepid" is a relative term. Salol melts at 104° F., and it would not be affected by such means. I do not believe the temperature of the roots of teeth is raised under any circumstance to that degree when hot fluids are taken into the mouth.

*Dr. McQuillen.*—I have been using salol, and have been perfectly delighted with it. In regard to what Dr. Roberts said, the salol is sealed in the tooth thoroughly, and if it did melt it would not make any difference; besides, I do not see how it would be possible to keep anything hot in the mouth long enough to melt the salol in a root. If it did, it would crystallize again when the temperature became normal. I have never had anything that has pleased me quite as much in root-filling.

*Dr. Curry.*—Would Dr. Kirk use salol where the apical foramen was unusually large?

*Dr. Kirk.*—I cannot recollect having used it in any such case as that; occasionally we have cases of open foramen, but they are not usual. I do not think I should hesitate to use it.

Dr. Mascort has described the use of salol in a case where caries had perforated the floor of the pulp-chamber, exposing the periosteum of the alveolus. If it would operate successfully in such a case, I see no reason why it would not in a case of open foramen, although it might be better to use a gutta-percha cone in connection with the salol.

*Dr. Deane.*—Would there be any danger in forcing salol through the apex where it was open?

*Dr. Kirk.*—I should not want to force anything through the apex. If I had to force anything through, I would prefer it to be salol to almost anything else of which I know; it must be used carefully and dexterously, so as to avoid any such action. That is an accident to be avoided in all canal operations.

*Dr. Deane.*—There are some roots through which we force medicine, for instance, in the treatment of a fistula.

*Dr. Kirk.*—I should prefer forcing a liquid medicament through in a case of that sort.

*Dr. Deane.*—After that would there be any danger in forcing salol through?

*Dr. Kirk.*—I should think not, if carefully used. Dr. Burchard uses a method somewhat different from that which I use. I use a Donaldson bristle to introduce the salol, first carrying the bristle to the apex of the root as nearly as I can; then with long pliers I carry the liquid up, and, having the broach in position as I introduce the salol, I slowly withdraw it so that I get the salol drawn into the space previously occupied by the instrument. There is a better chance by this method, I think, of carrying it to the apex. It can be readily pumped into a canal with a broach armed with cotton. It is a difficult matter to pump salol in liquid form vertically into a root-canal, but it will flow by capillary attraction, which carries it upward, so that we are reasonably certain that we have filled to the apex; that, of course, we never know absolutely until such a tooth is extracted and examined.

*Dr. Guilford.*—I have used salol in a way not mentioned by the previous speakers, and that is for the filling of root-canals in teeth about to be implanted. I recently used it in two cases of that kind, introducing the salt, by means of a fine heated wire, through the slightly enlarged apical foramen. It is so much easier of introduction in this manner than any other substance I have yet used for the same purpose.

*Dr. Kirk.*—Where I have used it in cases of filling roots of teeth for implantation, I have used a very small drill to enlarge the apical foramen and a hypodermic syringe to inject the salol into the tooth. By using a warm syringe and injecting the liquid salol into the root-canal from its apex, I believe it makes an excellent filling for such purposes. I have only had these cases of implanted teeth thus treated under observation for a short time.

*Dr. H. C. Register.*—I want to place myself on record as having used salol. I have used it as an antiseptic, also in connection with phosphate of zinc. I mix it in crystal form with phosphate of zinc, and insert it just as I would the plain phosphate of zinc in a canal. All of these cases, where salol has been used in connection with desiccation, which I have always, for a number of years past, carried out, have given me very good results.

There was one case, which occurs to me, of a gentleman with a very small mouth, where I had to treat a second inferior molar, and had to approach the pulp-chamber from the buccal wall. I tried to fill the tooth, desiccating it first, with salol and phosphate of zinc, but I never could make a perfectly comfortable tooth of it. After I found that salol could be melted so easily, I decided to seal it up permanently with melted salol. Keeping the tooth dry and desiccating it thoroughly, this was done. The apex of the root was open so that it would sometimes bleed right through from the canal, and yet the salol root-filling has worked very satisfactorily.

I operated on this case, which had been a very troublesome one, and it has now been six or eight months and there has not been the slightest trouble with it.

In all these cases I use no gutta-percha. I simply, after desiccation, fill the canal and a part of the pulp-chamber with the dry crystals, packed down into the canal as tightly as I can pack the material with an instrument, and then apply a small nerve instrument, warmed slightly, which, as soon as it touches the salol, liquefies it. As it becomes heated the material will pass into the most infinitesimal space, and almost as soon as the instrument is withdrawn the salol solidifies into a hard, crystalline mass.

In putrescent cases I use heated air and desiccate thoroughly. I then do not hesitate to melt the salol right into the canals and fill the tooth. I have no trouble with the cases at all.

In answer to Dr. Guilford, Dr. Register stated that he mixed the crystals of salol with the oxide of zinc before combining the latter with the phosphoric acid liquid.

*Dr. Kirk.*—I would like to ask if any one has used formol, or formalin, as it is also called. Dr. Mascort called my attention to the use of it in the St. Louis Hospital of Paris, I think, by a surgeon there, who had, in connection with the public clinic, a great many cases of alveolar abscess, etc., which were treated almost indiscriminately with formol. I had sent to me a bottle of formalin from Schering, of New York, and have been testing it in my work occasionally. At times I have found it a little too violent in its action, but by diluting it somewhat I get satisfactory results from it. Dr. Mascort, who suggested salol as a root-filling, was quite enthusiastic over the use of formol as a root-dressing for putrescent cases, and I am favorably impressed with its use in controlling putrescence and as an antiseptic. It is very penetrating. It is used in the hospital wards on the continent for spraying the walls, washing clothing, etc. The vapor makes a very efficient antiseptic; it is very penetrating when vaporized in a room, and a very small amount is destructive of germ-life. The odor somewhat resembles that of the allyl compounds,—onion, horseradish, mustard, oil, etc. It seems to me well worthy of investigation as a root-canal dressing.

*Dr. Louis Jack.*—Shortly after the use of formalin was suggested to me by Dr. Kirk, I made an application to a case which had been opened at the sea-shore during the summer. The dentist in charge of the case had made a mistake in the diagnosis, and assumed there was irritation in a certain tooth, and he destroyed the pulp and left the case open; the result was considerable disturbance in the apical region. The tooth had become so seriously affected that it would not allow closure; an ordinary cotton stopping would bring on, in a few hours, quite a serious disturbance, so I was obliged to leave it open and close it for a few hours only, and then open it. I employed a solution of formalin, diluting the ordinary solution to a five-per cent. solution, and on the second application I was able to close the tooth at once with gutta-percha, and in a few days afterwards filled it without any further disturbance.

I have also used it in cases where I formerly used hydronaphthol, but would warn any one against using it in a greater amount than five per cent. in pulp-treatment, on account of its irritating influence.

*Dr. Roberts.*—Is the practice to seal the formalin in the tooth, or is another dressing put in afterwards?

*Dr. Jack.*—In the case just reported, the only putrescent case in which I ever used it, I allowed it to remain in several minutes, then removed it, and applied aristol and gaultheria. I had previously applied aristol and gaultheria without any impression having been made.

*Dr. Darby.*—Does Dr. Jack think he would have had as good results if he had used hydronaphthol, or did he use it?

*Dr. Jack.*—I did not, as I would not consider it as active as aristol and gaultheria. I might state I have found formalin at ten per cent. quite an irritant.

*Dr. McQuillen.*—I would like to ask if any of the members have used Dr. Schreier's preparation, sodium potassium? I have been using it since September of last year, and the results have been so uniformly successful that I think with one exception it has never been necessary to again treat the tooth after thoroughly burning out or saponifying the putrescent matter in the pulp-chamber and canal. I have never had to use it a second time. One case I recall was sealed last September a year,—a lateral incisor in which the pulp-canal was positively filled with pus. It was the first case in which I used the Schreier preparation. That tooth has been sealed ever since, and that is only one of many cases.

Dr. Kirk gets the same effect, I believe, with sodium peroxide; but I have never used that, as this sodium potassium has helped me so fully. There is one objection to it. Unless you are careful not to use too large an amount, you are likely to have an explosion, the material going off like a fire-cracker, though no harm is done; only it causes a surprise.

*Dr. Curry.*—I have used it, but have lately abandoned it for sodium peroxide, as I like it better. It is easier to manipulate, and I get the same results.

*Dr. Kirk.*—There is a subject brought up by Dr. Burchard that has been passed over without discussion. If I remember correctly, in one of the early meetings of the Odontological Society there arose a discussion as to whether it was possible to have an abscess upon a vital tooth, and the meeting was considerably divided on that question. Dr. Burchard has called attention to a series of cases in which he had something similar to abscess on a vital tooth, the etiology of which he assigns to conditions of gouty or rheumatic diathesis.

I have had an exceptionally good opportunity, during the past ten days, to observe the whole period of inflammatory action of this character in the shape of an abscess in my own mouth. Two Sundays ago, and for some time afterwards, I was unable to get my jaws together, because of inflammation associated with a lower third molar. The inflammation was distinctly localized in the gum, on the buccal aspect of it. So far as I could determine by the sensation, by probing, etc., the inflammation was probably half-way down the side of the buccal aspect of the root, and accompanied with pain that almost incapacitated me from work for the time. It is such a pain as any one who has had an alveolar abscess knows a great deal about. There was only slight swelling. It was distinctly associated with general gouty or rheumatic symptoms. I passed a blunt probe around the gingival attachment, and found its margin absolutely perfect. There was no break in it, that I am morally certain of. In course of time the abscess broke, and pus discharged from it. There is now a pocket on the buccal side of the tooth, and I have no doubt it will be, in the course of time, the seat of a pyorrhœal condition, because that is a difficulty I have to continually combat.

I have the same condition repeated in an upper cuspid in my own mouth, and it may be of interest to Dr. Burchard to know that it also was coincident with a considerable manifestation of a rheumatic character. That is in my own individual case. The best way to know a thing is to know it by personal experience.

I have an exact duplicate of that case in the mouth of a lady patient of mine, excepting that the inflammation was associated with a lower second molar. I found exactly the same condition progressing there. The period through which the inflammation ran was about a week all told. I examined the gingival margin carefully, and found no break. It was a vital tooth. I, however, removed a large filling for the purpose of determining that. I was not satisfied with sensitiveness to thermal test. I wanted to test the responsiveness of the dentine, and I found a vital pulp, and still an abscess formed on the buccal side of the tooth. There is now a large pocket extending from the gingival margin almost to the apex of the root, discharging pus freely. It has been washed out and treated, but still there is an exudation.

I report these two cases because they have both been under my observation, and one of them will, I hope, continue to be for a number of years to come; and I believe there will be a record of pyorrhœa following this acute inflammatory outbreak unless that condition is cured in the mean time.

It seems to me that in these cases we have almost certainly the original lesion which induces the pyorrhœal condition. I do not maintain that what I have described is the only source of pyorrhœa, but I strongly suspect it to be the origin of one variety of it.

*Dr. Deane.*—I had a case a few days since in which the condition of pyorrhœa is very apparent: a discharge of pus in connection with the lower first molar on the right side, and the bicuspid on the left. I found on the lingual side of the lower right central a condition that led me to believe there was pus there, but no opening in the tissue leading down to the pus collected around the root of the tooth. I filed a broach off very thin, and passed that down around the tooth until I could feel the process, but I could not come in contact with the pus; but by opening up the sac from the lingual side I got quite a discharge of pus, and found I could pass a probe in right to the surface of the root. I believe that pus formed there without any possible chance of an opening around the cervical border of the tooth. I have placed the patient under the treatment recommended by Dr. Peirce, and shall watch the case carefully from time to time. I believe the tooth to be a vital one. No cavity in it at all; it responds to all the tests, heat and cold, etc.

Dr. Truman said he was somewhat sceptical on the point.

*Dr. Cryer.*—I do not see why we could not have such abscesses. The tooth can be alive and have an abscess in the immediate neighborhood. An abscess connected with a dead tooth-pulp would be, generally speaking, a dental alveolar abscess; that, of course, would involve the apex of the root. I do not see, however, why we could not have a live tooth and an abscess along the alveolar border.

*Dr. Gaskill.*—I had a case in which I got myself into trouble by a condition of that kind; a patient presented herself having a fistula between the first molar and second bicuspid, and I could not determine from which tooth the trouble arose. Both seemed somewhat sore and responded to the tests applied; finally determining to try the molar, I drilled into a vital pulp; I then drilled into the second bicuspid and found a vital pulp there, and had to destroy the pulp in the first molar, but was able to save the second bicuspid. The fistula was healed by systemic treatment. Afterwards the patient presented herself with a similar condition on the other side of the mouth.

*Dr. Truman.*—I question if proper discrimination is made between vitality of the roots; there may be vitality in one root, or even in two roots in the superior molars, and yet one be entirely dead; that very often occurs. In that dead portion of pulp abscess will occur; sooner or later pathogenic germs will find their way into that point.

When any one talks about an abscess at the root of the tooth without an opening, or that the tooth is entirely vital, I should want to know whether all the roots are vital. One may be dead and the other fully vital.

Care should be used in making this discrimination. I have no belief that abscess could occur near the apex of the tooth, and the tooth, or that root, retain its vitality.

*Dr. Kirk.*—In my own mouth there is not a dead pulp anywhere.

*Dr. Truman.*—Have you taken the tooth out?

*Dr. Kirk.*—No, I have it there now, and know that the pulp is alive. The tooth has no cavity in it.

Dr. Roberts cited a case of abscess on apparently sound teeth.

Dr. Curry described a case where he had drilled into a supposed dead pulp in order to treat an apical abscess with fistula, and had found the pulp entirely alive.

Dr. Jack mentioned having drilled into what he supposed was a dead pulp, but found it entirely alive, and said that twenty years ago he would not have believed as he now does, but he has seen so many cases that he is confident they do exist more commonly than some suppose.

*Dr. Register.*—Some years ago, before Dr. Peirce drew the attention of the profession to the fact that pyorrhœa alveolaris had some connection with rheumatism or gout, I knew that I had rheumatism. I also knew that I had erosion of the teeth, and I knew I had what was called Riggs's disease of the gums.

Several years ago, in connection with a lateral incisior which I still have in my mouth, a small abscess formed on the root about half-way between the gingival border and the apex. It was extremely painful, and it struck me as being singular that such a condition should exist, for I felt sure the pulp was alive. I never had had trouble with it to any extent, other than that it was a little loose. I tried to open the abscess from the cervical margin, passing an instrument up the line of the tooth-root, and was unable to reach it. I then slipped a lancet into it through the gum, and a small quantity of pus flowed out; since then there has been a recession of the gum up to the point where the pocket was formed. I am quite positive there was previously no opening in the gum margin, because I examined it with a mirror and instrument, and could see perfectly what I was doing. The pulp in that tooth is still alive.

Adjourned.

George D. B. Darby,
*Secretary.*

